# Prognostic implications of surrogate markers of atherosclerosis in low to intermediate risk patients with Type 2 Diabetes

**DOI:** 10.1186/1475-2840-11-101

**Published:** 2012-08-18

**Authors:** Kui-Kai Lau, Yuen-Kwun Wong, Yap-Hang Chan, Kai-Hang Yiu, Kay-Cheong Teo, Leonard Sheung-Wai Li, Shu-Leong Ho, Koon-Ho Chan, Chung-Wah Siu, Hung-Fat Tse

**Affiliations:** 1 Division of Neurology, Department of Medicine, Queen Mary Hospital, The University of Hong Kong, Hong Kong SAR, China; 2 Research Center of Heart, Brain, Hormone and Healthy Aging, Li Ka Shing Faculty of Medicine, The University of Hong Kong, Hong Kong SAR, China; 3 School of Public Health, The University of Hong Kong, Hong Kong SAR, China; 4 Division of Cardiology, Department of Medicine, Queen Mary Hospital, The University of Hong Kong, Hong Kong SAR, China; 5 Division of Rehabilitation Medicine, Department of Medicine, Tung Wah Hospital, The University of Hong Kong, Hong Kong SAR, China

**Keywords:** Vascular markers of atherosclerosis, Type 2 diabetes mellitus

## Abstract

**Background:**

Type 2 diabetes mellitus (T2DM) patients are at increased risk of developing cardiovascular events. Unfortunately traditional risk assessment scores, including the Framingham Risk Score (FRS), have only modest accuracy in cardiovascular risk prediction in these patients.

**Methods:**

We sought to determine the prognostic values of different non-invasive markers of atherosclerosis, including brachial artery endothelial function, carotid artery atheroma burden, ankle-brachial index, arterial stiffness and computed tomography coronary artery calcium score (CACS) in 151 T2DM Chinese patients that were identified low-intermediate risk from the FRS recalibrated for Chinese (<20% risk in 10 years). Patients were prospectively followed-up and presence of atherosclerotic events documented for a mean duration of 61 ± 16 months.

**Results:**

A total of 17 atherosclerotic events in 16 patients (11%) occurred during the follow-up period. The mean FRS of the study population was 5.0 ± 4.6% and area under curve (AUC) from receiver operating characteristic curve analysis for prediction of atherosclerotic events was 0.59 ± 0.07 (*P = 0.21*). Among different vascular assessments, CACS > 40 had the best prognostic value (AUC 0.81 ± 0.06, *P < 0.01*) and offered significantly better accuracy in prediction compared with FRS (*P = 0.038* for AUC comparisons). Combination of FRS with CACS or other surrogate vascular markers did not further improve the prognostic values over CACS alone. Multivariate Cox regression analysis identified CACS > 40 as an independent predictor of atherosclerotic events in T2DM patients (Hazards Ratio 27.11, 95% Confidence Interval 3.36-218.81, *P = 0.002*).

**Conclusions:**

In T2DM patients identified as low-intermediate risk by the FRS, a raised CACS > 40 was an independent predictor for atherosclerotic events.

## Background

Patients with type 2 diabetes mellitus (T2DM) are at increased risk of developing adverse atherosclerotic events including acute coronary syndrome (ACS) and ischemic stroke [1-3]. Various risk assessment algorithms, for example the Framingham Risk Score (FRS) and Systematic Coronary Risk Evaluation (SCORE), aim to predict the likelihood of developing cardiovascular events [4-6]. Some of these scores have also been recalibrated based on the ethnic differences in cardiovascular risk profile and incidences of cardiovascular diseases (CVD). Nonetheless only the Framingham Risk Score has been calibrated for use in the Chinese population [7]. Although these algorithms are considered a useful tool for risk stratification in the general population, they do not have sufficient power and lack accuracy in patients with T2DM [8-10]. Furthermore, whilst current guidelines provide clear recommendations on initiating anti-platelet therapy for primary prevention of CVD in diabetic patients with high cardiovascular risk, not those at low risk, there are no recommendations for those categorized as intermediate risk [11].

Surrogate markers of atherosclerosis, including brachial artery flow-mediated dilatation (FMD), carotid artery atheroma burden, ankle-brachial index (ABI), arterial stiffness and computed tomography coronary artery calcium score (CACS) have been increasingly used for the prediction of cardiovascular events [12-16]. Prior studies have demonstrated that such markers improve risk stratification of CVD in the general population when used in conjunction with the FRS [12,14,15,17-22]. Recent studies also support the use of carotid intima-media thickness (IMT) and CACS in conjunction with traditional risk scores to improve risk stratification in diabetics asymptomatic for CVD [16,23]. The prognostic values of surrogate markers of atherosclerosis in T2DM patients deemed low-intermediate risk is nevertheless currently unknown. The purpose of this prospective study was to compare the prognostic values of the FRS with a range of commonly used surrogate markers of atherosclerosis, used alone and in combination with the FRS, in Chinese T2DM patients identified as low-intermediate risk.

## Methods

### Subjects

Consecutive Chinese patients with T2DM as defined by the WHO criteria were prospectively recruited from the Medical Outpatient Clinics from July 2005 to December 2006 [24,25]. Patients calculated to be at high risk (≥20%) of developing a cardiovascular event within 10 years based on the recalibrated FRS for Chinese were excluded [7]. Patients with a history of prior athero-thrombotic events (including ACS, ischemic stroke, acute limb ischemia), stable angina, symptomatic peripheral vascular disease, creatinine level >220 μmol/L, severe hepatic disease, malignancy or connective tissue diseases were also excluded. A total of 151 patients with T2DM were thus eligible for this study. We obtained approval from the local institutional review board and all subjects gave written informed consent.

### Study design

Baseline demographic data, cardiovascular risk factors and cardiovascular medication at the time of recruitment were documented. Cardiovascular risk factors including tobacco smoking, hypercholesterolemia, hypertension and family history of CVD diagnosed in first-degree relatives before 55 years of age were assessed. Hypertension was defined as either resting systolic or diastolic blood pressure ≥140 or ≥90 mmHg respectively, recorded at two different clinical visits or the prescription of anti-hypertensive medication [26]. Hypercholesterolemia was defined as fasting total plasma cholesterol ≥5.1 mmol/L or the prescription of lipid-lowering medication [27]. Smoking status was recorded as ever-smoker (past or current) or non-smoker.

Anthropometric measurements including body weight, height and waist-hip circumference ratio (WHR) were performed. Body-mass index (BMI) was calculated as kg/m^2^. Systolic and diastolic blood pressures were measured and fasting blood samples were obtained to measure serum levels of glucose, hemoglobin A1c (HbA1c), total cholesterol, triglyceride, low-density lipoprotein-cholesterol (LDL-C), high-density lipoprotein-cholesterol (HDL-C) and creatinine. Glomerular filtration rate (GFR) was calculated using the Modification of Diet in Renal Disease Study equation [28].

### Vascular assessments

Vascular ultrasound examination for brachial endothelial function, carotid IMT and presence of carotid plaque was evaluated through a standard B-mode ultrasound examination with the use of a 7.5 MHz linear array transducer and a high resolution ultrasound system (Agilent Sonos 5500, Philips, Andover, Massachusetts, USA) as described previously [18]. Measurements of ABI and arterial stiffness were performed using a commercially available device based on the oscillometric method (VP-2000, Colin Corporation, Komaki, Japan) [25]. Arterial stiffness was represented by brachial to ankle pulse wave velocity (baPWV). This method has previously been validated and closely correlates with aortic PWV [29].

A single experienced operator blinded to the status of the study subjects performed all vascular ultrasound examinations. Another experienced operator, also blinded to the status of study subjects, operated on VP-2000 and obtained ABI and arterial stiffness parameters.

#### Brachial endothelial function

Patients were studied in the fasting state and vasoactive medications were withheld for 12 hours prior to the scans. Longitudinal brachial artery diameter was obtained at rest, and then during FMD, induced by inflation of a pneumatic tourniquet placed around the forearm to a pressure of 50 mmHg above systolic blood pressure for 5 minutes. The cuff was then released and serial imaging of the brachial artery was recorded for 5 minutes. FMD was defined as the percentage change in brachial artery diameter between 1 minute following cuff deflation and that on the baseline scan. All digital images were stored on optical diskettes for subsequent off-line analysis using a computer workstation (EchoPAC, GE Medical, Wisconsin, USA). A single operator measured the brachial artery diameter and an average value from three consecutive measurements was calculated. The intra-observer correlation coefficient for FMD was 0.90 (2 repeated measurements in 20 randomly chosen subjects).

#### Carotid intima-media thickness and plaque

Carotid IMT was determined by measuring manually the distance between the lumen-intima and media-adventia border of the vascular wall using electronic calipers. Each ultrasonic scan was performed in the anterior, lateral and posterior projections of the right and left carotid arteries. Three IMT measurements were made on the near and far wall of the common carotid arteries, carotid bifurcation and internal carotid arteries. The mean maximum IMT (mmIMT) was used for analysis and was calculated by averaging the values of maximum IMT from 12 pre-selected segments of the carotid arteries. Presence of carotid plaque was defined as an endoluminal protrusion of the arterial lumen of at least 0.5 mm or 50% of the surrounding IMT value or demonstration of an IMT of >1.5 mm [30]. The intra-observer correlation coefficient for mmIMT was 0.97 (2 repeated measurements in 20 randomly chosen subjects).

#### Ankle-brachial index and arterial stiffness

Measurements of ABI and arterial stiffness were performed with subjects in the supine resting position in a quiet and temperature-controlled room. Pneumatic pressure cuffs with oscillometric pressure sensors were wrapped tightly around both arms and both ankles. Electrocardiographic electrodes were attached to both wrists and a phonocardiogram was placed at the left second intercostal space, at the margin of the sternum. After ensuring that patients had rested for 15 minutes, fully automatic data acquisition began. Pressure waveforms of the brachial and posterior tibial arteries were recorded. Based on the height of the patient, the device estimated the path lengths from the brachial artery to the posterior tibial artery. BaPWV was calculated as the path length divided by the corresponding time interval (cm/s). The right and left baPWV were averaged and the resulting value selected as the representative baPWV.

The pneumatic cuffs over both arms and ankles enabled simultaneous measurement of systolic blood pressure at each limb. The right-and left-sided ABI were calculated as the ankle systolic blood pressure divided by the brachial systolic blood pressure measured from the right and left side, respectively. The right and left ABI were averaged and the resulting value selected as the representative ABI.

The intra-observer correlation coefficient for ABI was 0.85 and the intra-observer correlation coefficient for baPWV was 0.98 (2 repeated measurements in 20 randomly chosen subjects).

#### Computed tomography coronary artery calcium score

All subjects underwent computed tomography imaging of the coronary arteries using a 64 slice MDCT (Lightspeed, VCT, GE Healthcare, USA) as described previously [18,31]. All scans were performed with subjects in the supine position, and included regions from the arch of the aorta to the fundus of the heart. Prospective electrocardiogram-gated cardiac scan was obtained with the following scan variables: rotation time = 0.35 s, slice thickness = 2.5 mm; 120 kV; 250 mA; trigger delay = 70% R-R interval. Patients were instructed to breath hold for 30s during scanning.

The acquired MDCT images were reviewed at the post-processing image workstation (Advantage windows 4.02, GE Healthcare). Complete data were available from all scans, with no mis-registration of slices due to artifacts of motion, respiration, or asynchronous electrocardiographic triggering. To ensure continuity and consistency of interpretation of calcium scores, two expert investigators, who were unaware of subject’s clinical status, analyzed all scans. The inter-observer and intra-observer variability correlation coefficients of CACS measurements were 0.92 and 0.91, respectively.

Measurement of CACS was performed using a commercially available software “smart score” (GE Healthcare) using the threshold option set for pixels >130 Hounsefiled units and expressed in Agaston units. CACS was calculated as the sum of calcium scores in the left main coronary artery, left anterior descending artery, left circumflex coronary artery, right coronary artery, and posterior descending artery.

### Clinical outcome

All patients were followed-up in our clinic every 3–4 months. Clinical data of all patients were retrieved from the medical records and subsequently during the most recent clinic visit. An adverse atherosclerotic event was defined as ACS, ischemic stroke, new onset symptomatic peripheral vascular disease, death due to ACS or ischemic stroke or symptom driven revascularization procedures of the carotid, coronary or peripheral arteries.

### Statistical analysis

Power calculation was performed based on the prediction values of coronary artery calcium score for atherosclerotic events in patients with type 2 diabetes. Using an alpha of 0.05 and total sample size of 151 would give a power of 80%.

Data were expressed as mean ± standard deviation for continuous variables and proportions for categorical variables. Baseline characteristics were compared between groups using Student’s *t*-test or Chi-squared test, as appropriate. Pearson’s correlation was used to measure the correlations between vascular assessment variables. Area under receiver operating curves (AUC) were calculated and the cut-off values of the vascular assessments with optimal sensitivity and specificity were obtained. These cut-off values were then used in subsequent analysis to determine the prognostic role of these surrogate markers in the prediction of atherosclerotic events. Chi-squared test was also used to compare the predictive accuracy between FRS and vascular markers in atherosclerotic event prediction. The associations between vascular assessment parameters, categorized by AUC cut-off values, and event-free survival were evaluated using Cox regression models with adjustment for potential confounding variables. Only parameters with *P < 0.1* in uni-variate analysis were entered into a multi-variate model to identify the independent predictors for atherosclerotic events. Cumulative event rates were estimated using the Kaplan-Meier method and compared using log-rank test.

All statistical analyses were performed using the statistical software SPSS for Windows (Version 15.0, SPSS, Chicago, USA) and STATA for Windows (Version 11.2, STATA Corp., College Station, TX). A *P* value *<0.05* was considered statistically significant.

## Results

### Clinical characteristics

Clinical characteristics of the study population are summarized in Table 1. Their average age was 60.9 ± 10.0 years and 40% were men. Their mean duration of DM was 15.2 ± 7.5 years, 60% patients had hypertension, 56% had hypercholesterolemia, 18% were ever-smokers and 17% had a family history of CVD. The mean HbA1C was 7.7 ± 1.2%, 16% of patients had retinopathy and the average GFR was 84 ± 22 ml/min/1.73 m^2^. The mean FRS of the study population was 5.0 ± 4.6%. Results of the vascular assessment parameters are shown in Table 1.

**Table 1:** Clinical characteristics of the study population

**Characteristic**	**All**	**With Atherosclerotic Event**	**Without Atherosclerotic Event**	**P-value**
	(N = 151)	(N = 17)	(N = 134)	
Age, years	60.9 ± 10.0	64.9 ± 10.1	60.4 ± 9.9	0.079
Males, n (%)	61 (40)	10 (59)	51 (38)	0.10
Hypertension, n (%)	89 (60)	8 (47)	81 (62)	0.24
Hypercholesterolemia, n (%)	80 (56)	9 (60)	71 (55)	0.71
Ever-smokers, n (%)	27 (18)	6 (35)	21 (16)	0.047
Family history of CVD, n (%)	25 (17)	2 (12)	23 (17)	0.57
Duration of DM, years	15.2 ± 7.5	14.1 ± 7.8	15.3 ± 7.5	0.54
BMI, kg/m^2^	25.6 ± 4.1	23.8 ± 3.3	25.8 ± 4.1	0.061
WHR	88.5 ± 10.4	85.6 ± 8,5	88.9 ± 10.6	0.23
Systolic blood pressure, mmHg	140 ± 21	143 ± 21	139 ± 21	0.46
Diastolic blood pressure, mmHg	78 ± 9	76 ± 9	78 ± 9	0.49
Fasting blood glucose, mmol/L	7.5 ± 2.0	8.0 ± 2.9	7.5 ± 1.9	0.38
HbA1c, %	7.7 ± 1.2	7.7 ± 1.1	7.7 ± 1.3	0.94
Triglyceride, mmol/L	1.5 ± 1.2	1.5 ± 0.8	1.6 ± 1.2	0.77
Total cholesterol, mmol/L	4.9 ± 0.9	5.0 ± 1.1	4.9 ± 0.9	0.69
LDL-C, mmol/L	2.8 ± 0.8	3.0 ± 0.8	2.8 ± 0.7	0.46
HDL-C, mmol/L	1.4 ± 0.4	1.4 ± 0.3	1.4 ± 0.4	0.73
GFR, ml/min/1.73 m^2^	84 ± 22	74 ± 27	85 ± 21	0.059
Beta-blocker, n (%)	29 (21)	2 (13)	27 (22)	0.40
Calcium channel blocker, n (%)	50 (36)	4 (25)	46 (37)	0.35
ACEI/ARB, n (%)	44 (31)	6 (38)	38 (30)	0.56
Aspirin, n (%)	8 (6)	1 (6)	7 (6)	0.92
Statin, n (%)	30 (21)	5 (31)	25 (20)	0.31
Oral hypoglycemic agent, n (%)	115 (82)	13 (81)	102 (82)	0.92
Insulin, n (%)	24 (17)	2 (13)	22 (18)	0.60
Retinopathy, n (%)	23 (16)	3 (19)	20 (16)	0.76
Urine albumin g/l	0.07 ± 0.2	0.1 ± 0.3	0.1 ± 0.2	0.60
FRS, %	5.0 ± 4.6	5.9 ± 5.0	4.9 ± 4.5	0.41
FMD, %	2.8 ± 3.8	1.7 ± 3.9	2.9 ± 3.7	0.22
mmIMT, mm	0.9 ± 0.2	1.1 ± 0.3	0.9 ± 0.2	0.047
Carotid plaque, n (%)	85 (57)	12 (71)	73 (55)	0.22
ABI	1.1 ± 0.1	1.0 ± 0.2	1.1 ± 0.1	0.080
baPWV, cm/s	1752 ± 384	1897 ± 373	1732 ± 383	0.20
CACS	148 ± 353	374 ± 341	120 ± 346	0.011

Abbreviations: ABI = ankle brachial index; ACEI = angiotensin converting enzyme inhibitor; ARB = angiotensin receptor blocker; baPWV = brachial-ankle pulse wave velocity; BMI = body mass index; CACS = coronary artery calcium score; CVD = cardiovascular disease; DM = diabetes mellitus; FMD = flow mediated dilatation; FRS = Framingham Risk Score; GFR = glomerular filtration rate; HbA1c = haemoglobin A1c; HDL = high-density lipoprotein cholesterol; LDL-C = low-density lipoprotein cholesterol; mmIMT = mean maximum intima-media thickness; WHR = waist-hip circumference ratio.

### Relationship between Framingham risk score and vascular assessment parameters

Table 2 shows the correlation coefficients between FRS and various vascular assessments. There was a modest but significant correlation between FRS and mmIMT, baPWV and CACS (all *P < 0.01*). CACS also correlated well with mmIMT, ABI and baPWV (all *P < 0.05*) and mmIMT correlated well with CACS, ABI and baPWV (all *P < 0.01*). FMD showed no correlation with the FRS nor with the other vascular markers.

**Table 2:** Pearson correlation coefficients between FRS and vascular assessment parameters

	**FMD**	**mmIMT**	**ABI**	**baPWV**	**CACS**
mmIMT	−0.07				
ABI	0.02	−0.42**			
baPWV	0.10	0.36**	−0.22		
CACS	0.03	0.35**	−0.29*	0.49**	
FRS	−0.06	0.31**	−0.17	0.34**	0.33**

Abbreviations as in Table 1.

*P < 0.05, **P < 0.01.

### Clinical outcomes

During a mean follow-up of 61 ± 16 months (range 3–75 months), 17 atherosclerotic events including 1 cardiovascular death, 1 ACS, three ischemic stroke, 2 new onset peripheral vascular disease and 11 percutaneous coronary interventions were observed in 16 patients (11% of the study population). Elective percutaneous coronary interventions were performed at a mean of 8 ± 4 months following CT examination. All percutaneous coronary interventions were performed for treatment of symptomatic coronary artery disease and / or based on the presence of inducible ischemia detected by functional assessment rather than the results of CACS.

Table 1 shows the clinical characteristics and vascular marker parameters in patients with and without atherosclerotic event. Patients with atherosclerotic events were more likely to be smokers (35% versus 16%, *P = 0.047*). There was also a trend for patients with atherosclerotic events to be older (64.9 ± 10.1 years versus 60.4 ± 9.9 years, *P = 0.079*), have a lower BMI (23.8 ± 3.3 kg/m^2^ versus 25.8 ± 4.1 kg/m^2^, *P = 0.061)* and a lower GFR (74 ± 27 ml/min/1.73 m^2^ versus 85 ± 21 ml/min/1.73 m^2^, *P = 0.059*). There were nonetheless no significant differences between the two groups in terms of gender, proportions with hypertension or hypercholesterolemia, duration of DM, HbA1c or use of medication.

Patients with atherosclerotic events had a significantly greater carotid artery mmIMT (1.1 ± 0.3 mm versus 0.9 ± 0.2 mm, *P = 0.047*) and CACS (374 ± 341 versus 120 ± 346, *P = 0.011*) than those without atherosclerotic events. No significant differences in FRS, brachial artery FMD, prevalence of carotid plaque, ABI or baPWV were observed between the two groups.

### Prognostic values of vascular assessments

ROC curves were constructed to obtain the prognostic values and optimal cut-off values of the FRS as well as different vascular assessment parameters (Table 3). All of these parameters had good negative predictive values (90-100%) but poor positive predictive values (14-57%). The FRS had an AUC of 0.59 ± 0.07 (*P = 0.21*). In contrast, of all the vascular assessment parameters tested, CACS >40 had the best predictive power for an atherosclerotic event (AUC 0.81 ± 0.06, *P < 0.01*) followed by carotid artery mmIMT > 1.07 mm (AUC 0.67 ± 0.07, *P = 0.02*).

**Table 3:** Prognostic values of FRS and vascular assessments according to specified cut-off values

**Markers**	**AUC (SE)**	**Cut-off values**	**Sensitivity, % (95%CI)**	**Specificity, % (95 % CI)**	**PPV, %**	**NPV, %**
FRS	0.59 ± 0.07	>2.56 %	82.4 (56.6-96.2)	47.8 (39.1-56.6)	16.7	95.5
FMD	0.59 ± 0.08	≤1.12 %	52.9 (27.8-77.0)	72.4 (64.0-79.8)	19.6	92.4
mmIMT	0.67 ± 0.07*	>1.07 mm	41.2 (18.4-67.1)	85.7 (78.6-91.2)	26.9	91.9
Carotid plaque	0.58 ± 0.06	-	70.6 (44.0-89.7)	45.11 (36.5-54.0)	14.1	92.3
ABI	0.64 ± 0.10	≤0.98	33.3 (9.9-65.1)	95.9 (88.5-99.1)	57.1	89.7
baPWV	0.63 ± 0.09	>1467 cm/s	100.0 (69.2-100.0)	26.4 (16.7-38.1)	15.9	100
CACS	0.81 ± 0.06**	>40	92.9 (66.1-99.8)	67.3 (57.8-75.8)	26.0	98.7

Abbreviations as in Table 1 and AUC = area under curve; SE = standard error; CI = confidence interval; PPV = positive predictive value; NPV = negative predictive value*P < 0.05, **P < 0.01.

**Figure 1:**
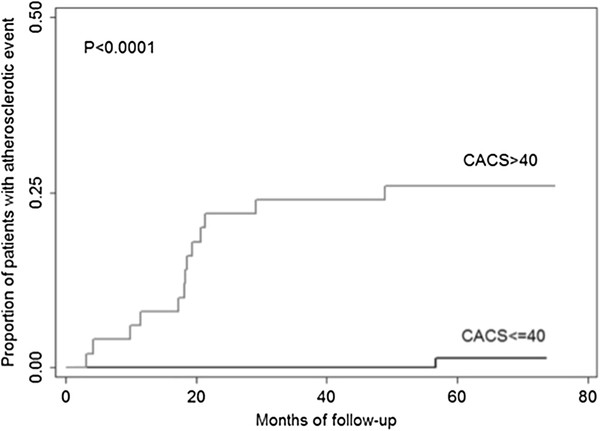
Kaplan-Meier curve for the development of atherosclerotic events in patients with CACS > 40 or CACS ≤ 40.

Uni-variate analysis identified age, male sex, BMI, WHR, hypertension, FMD ≤1.12%, mmIMT >1.07 mm, ABI ≤0.98 and CACS >40 as positive predictors for an atherosclerotic event (all *P < 0.1*). Multi-variate Cox regression analysis revealed that mean ABI ≤0.98 (Hazards ratio (HR) 7.17, 95% CI (confidence interval) 1.63-31.52, *P = 0.009*) and CACS >40 (HR 27.11, 95% CI 3.36-218.81, *P = 0.002*) were independent predictors of an atherosclerotic event (Table 4). There was also a trend for mmIMT >1.07 mm as an independent predictor of an atherosclerotic event (HR 2.76, 95% CI 0.90-8.46, *P = 0.077*). In Kaplan-Meier analysis, a raised CACS >40 was associated with the occurrence of an atherosclerotic event during follow-up (*P < 0.0001*, Figure 1).

**Table 4:** Multivariate Cox regression for atherosclerotic events in relation to vascular markers and risk factors

**Risk variable**	**mmIMT**		**ABI**		**CACS**	
	**HR (95 % CI)**	***P-*value**	**HR (95 % CI)**	**P-value**	**HR (95 % CI)**	**P-value**
mmIMT > 1.07 mm	2.76 (0.90-8.46)	0.077	-	-	-	-
ABI ≤ 0.98	-	-	7.17 (1.63-31.52)	0.009	-	-
CACS > 40	-	-	-	-	27.11 (3.36-218.81)	0.002
Age	1.05 (1.00-1.11)	0.079	1.03 (0.97-1.09)	0.33	1.01 (0.95-1.08)	0.70
Males	2.32 (0.79-6.78)	0.12	2.43 (0.81-7.25)	0.11	2.17 (0.64-7.41)	0.22
BMI	0.91 (0.67-1.24)	0.56	0.85 (0.63-1.15)	0.29	0.81 (0.58-1.12)	0.20
WHR	1.00 (0.89-1.12)	0.99	1.02 (0.91-1.14)	0.73	1.05 (0.93-1.19)	0.41
Hypertension	0.55 (0.20-1.51)	0.24	0.71 (0.25-2.00)	0.52	0.53 (0.18-1.60)	0.26

Abbreviations as in Tables 1, 2, 3.

Finally, we compared the predictive power of FRS for atherosclerotic events against other vascular assessment markers, alone and in combination with the FRS (Table 5). CACS had significantly greater AUC compared with FRS (AUC 0.81 versus 0.59, *P = 0.038*). There were no significant differences in AUC between other vascular markers and FRS (all *P > 0.05*). Combining CACS with FRS provided incremental benefit in risk prediction compared with FRS alone (AUC 0.77 versus 0.59, *P = 0.013*) but did not offer further benefit compared with CACS alone. Combination of FRS with other vascular markers also did not provide further incremental benefit.

**Table 5:** Comparison of the AUC of FRS alone and in combination with vascular assessment markers for atherosclerotic events

	**AUC (SE)**	**P-Value**
FRS	0.59 ± 0.07	-
FRS + FMD	0.62 ± 0.07	0.66
FRS + mmIMT	0.67 ± 0.07	0.34
FRS + carotid plaque	0.61 ± 0.07	0.77
FRS + ABI	0.67 ± 0.10	0.29
FRS + baPWV	0.66 ± 0.09	0.30
FRS + CACS	0.77 ± 0.06	0.013

Abbreviations as in Tables 1, 2, 3.

## Discussion

This study compared the diagnostic value of non-invasive markers of atherosclerosis, used alone and with the FRS, in the prediction of atherosclerotic events in Chinese T2DM deemed low-intermediate risk. The results of our study show that in Chinese T2DM patients of low-intermediate risk, 11% developed an atherosclerotic event during a mean follow-up of 61 ± 16 months. The FRS only had a modest accuracy in prediction of atherosclerotic events in our study. In contrast, among the different surrogate markers of atherosclerosis evaluated, a raised CACS > 40 offered the best prognostic value and was superior to FRS in risk prediction. It was also an independent predictor of a future atherosclerotic event. Although combination of CACS with FRS provided incremental benefit in risk prediction versus FRS alone, such a combination did not offer further prognostic value compared with CACS alone.

### Limitations of traditional risk scores

The FRS has been successful in prediction of CVD in general populations. The risk algorithm nonetheless lacks accuracy in patients with diabetes [4,5]. Studies using the FRS in different population groups have found that the FRS has both under- and over-estimated CVD risk in patients with DM [8-10]. Such an observation has been postulated to be related to a small number of diabetic patients in the original Framingham cohort (4% of 5573 subjects) from which the equation was derived. Glycemic control as well as duration of diabetes are also considered important parameters in determining CVD risk and should be included in risk algorithms [8]. Among Chinese, the FRS also overestimates cardiovascular risk, leading to a recent recalibration to suit the Chinese population [7]. Although this has led to improved risk estimation, recent studies have further identified limitations of such a model due to the greater prevalence of stroke than coronary heart disease in China [32]. In this study, the average FRS of our study population was only 5.0 ± 4.6% for prediction of cardiovascular events in 10 years but up to 11% (16 out of 151) of patients developed an adverse cardiovascular event within a mean follow-up period of 61 ± 16 months, thus highlighting the limitations of the FRS.

### Use of vascular markers in improving cardiovascular risk stratification

In view of the FRS’s drawbacks, further strategies to improve risk stratification should be implemented. Previous studies have identified that use of non-invasive surrogate markers of atherosclerosis including brachial artery FMD, carotid atheroma burden, ABI, arterial stiffness and CACS improve CVD risk stratification in the general population [12,14,15,17-22]. Fewer such studies have been performed in a population limited to patients with T2DM. Recent studies have shown that combination of the FRS with carotid artery IMT provides greater predictive power of cardiovascular events compared with FRS alone [23,33], but such a benefit was not observed when combining baPWV with FRS [23]. Similarly the PREDICT study provided evidence that measurement of the CACS was a powerful predictor of cardiovascular events in asymptomatic patients with T2DM and was able to improve risk prediction as calculated by the United Kingdom Prospective Diabetes Study risk model [16].

Amongst commonly used non-invasive surrogate markers of atherosclerosis, a raised CACS was determined to have the best prognostic value in this study with a high negative predictive value of 98.7%. This finding is consistent with similar studies that also showed CACS to be superior to other markers for CVD prediction in the general population [22,34]. It has been suggested that the superiority of CACS in coronary heart disease prediction is likely related to the direct measurement of atheroma burden in the vasculature bed of interest [22]. Nonetheless despite a range of end points related to atherosclerotic disease being used in this study, CACS remained superior to other vascular markers. The significant correlations of CACS with mmIMT, ABI and arterial stiffness suggests that CACS is not only a reflection of atheroma burden in the coronary arteries but of other vasculature beds including the carotid arteries and peripheral vasculatures.

The most recent international guidelines provide clear recommendations for initiation of antiplatelet therapy in diabetic patients considered to be at high cardiovascular risk (men >50 years or women >60 years with one additional major cardiovascular risk factor) and does not recommend such therapy for those at low risk (men <50 years or women <60 years with no major cardiovascular risk factors) [11]. Nonetheless the prescription of anti-platelet agents remains a matter of clinical judgment for those at intermediate risk e.g. young patients with cardiovascular risk factors or older patients with no risk factors [11]. Our results show that screening of atherosclerotic burden with CACS may identify a high risk subgroup of diabetic patients who have been classified as intermediate risk using FRS. This group of patients may benefit from more aggressive preventive measures, including lipid lowering and anti-platelet therapy for CVD. Our results echo those from previous studies that have also demonstrated CACS as a powerful predictor of CVD and all-cause mortality as well as enhancing risk prediction in asymptomatic diabetic patients [16,26,35,36].

### Limitations

In this prospective observational study, there was a small sample size that of only 151 Chinese patients with T2DM resulting in 17 events. Further large scale studies replicated in other ethnic populations and using other risk algorithms are needed to confirm our findings. In addition, there is increasing interest in coronary artery plaque location and morphology as detected by contrast CT coronary angiogram but this was not assessed in the current study [35,37]. Finally, whilst serial monitoring of surrogate markers of atherosclerosis in diabetics have recently been studied to delineate the change in vascular parameters with treatment and time, this was not performed in our study [36,38].

## Conclusions

In this small study, detection of CACS > 40 was an independent predictor for atherosclerotic events in T2DM patients identified as low-intermediate risk by the FRS.

## Competing interest

The author(s) declare that they have no competing interests.

## Authors’ contribution

KKL carried out the study and wrote the manuscript; YKW retrieved the follow-up data & performed the statistical analysis; YHC, KHY & KCT recruited the subjects & performed the vascular assessments; LSWL, SLH, KHC & CWS supervised the study; and HFT supervised the study design as well as writing of the manuscript. All authors read and approved the final manuscript.

## References

[B1] Rytter L Troelsen S Beck-Nielsen H Prevalence and mortality of acute myocardial infarction in patients with diabetes Diabetes Care 1985 8 3 230 234 10.2337/diacare.8.3.230 4006657

[B2] Kannel WB McGee DL Diabetes and cardiovascular disease. The Framingham study JAMA 1979 241 19 2035 2038 10.1001/jama.1979.03290450033020 430798

[B3] Lavy S Melamed E Cahane E Carmon A Hypertension and diabetes as risk factors in stroke patients Stroke 1973 4 5 751 759 10.1161/01.STR.4.5.751 4751086

[B4] D'Agostino RB Grundy S Sullivan LM Wilson P Validation of the Framingham coronary heart disease prediction scores: results of a multiple ethnic groups investigation JAMA 2001 286 2 180 187 10.1001/jama.286.2.180 11448281

[B5] Ramachandran S French JM Vanderpump MP Croft P Neary RH Using the Framingham model to predict heart disease in the United Kingdom: retrospective study BMJ 2000 320 7236 676 677 10.1136/bmj.320.7236.676 10710574 PMC27308

[B6] Conroy RM Pyorala K Fitzgerald AP Sans S Menotti A De Backer G De Bacquer D Ducimetiere P Jousilahti P Keil U Estimation of ten-year risk of fatal cardiovascular disease in Europe: the SCORE project Eur Heart J 2003 24 11 987 1003 10.1016/S0195-668X(03)00114-3 12788299

[B7] Liu J Hong Y D'Agostino RB Wu Z Wang W Sun J Wilson PW Kannel WB Zhao D Predictive value for the Chinese population of the Framingham CHD risk assessment tool compared with the Chinese multi-provincial cohort study JAMA 2004 291 21 2591 2599 10.1001/jama.291.21.2591 15173150

[B8] Coleman RL Stevens RJ Retnakaran R Holman RR Framingham, SCORE, and DECODE risk equations do not provide reliable cardiovascular risk estimates in type 2 diabetes Diabetes Care 2007 30 5 1292 1293 10.2337/dc06-1358 17290036

[B9] McEwan P Williams JE Griffiths JD Bagust A Peters JR Hopkinson P Currie CJ Evaluating the performance of the Framingham risk equations in a population with diabetes Diabet Med 2004 21 4 318 323 10.1111/j.1464-5491.2004.01139.x 15049932

[B10] van der Heijden AA Ortegon MM Niessen LW Nijpels G Dekker JM Prediction of coronary heart disease risk in a general, pre-diabetic, and diabetic population during 10 years of follow-up: accuracy of the Framingham, SCORE, and UKPDS risk functions: The Hoorn Study Diabetes Care 2009 32 11 2094 2098 10.2337/dc09-0745 19875606 PMC2768197

[B11] Standards of medical care in diabetes--2012 Diabetes Care 2012 35 Suppl 1 S11 S63 22187469 10.2337/dc12-s011 PMC3632172

[B12] Yeboah J Folsom AR Burke GL Johnson C Polak JF Post W Lima JA Crouse JR Herrington DM Predictive value of brachial flow-mediated dilation for incident cardiovascular events in a population-based study: the multi-ethnic study of atherosclerosis Circulation 2009 120 6 502 509 10.1161/CIRCULATIONAHA.109.864801 19635967 PMC2740975

[B13] Ali YS Rembold KE Weaver B Wills MB Tatar S Ayers CR Rembold CM Prediction of major adverse cardiovascular events by age-normalized carotid intimal medial thickness Atherosclerosis 2006 187 1 186 190 10.1016/j.atherosclerosis.2005.09.003 16233899

[B14] Mitchell GF Hwang SJ Vasan RS Larson MG Pencina MJ Hamburg NM Vita JA Levy D Benjamin EJ Arterial stiffness and cardiovascular events: the Framingham Heart Study Circulation 2010 121 4 505 511 10.1161/CIRCULATIONAHA.109.886655 20083680 PMC2836717

[B15] Fowkes FG Murray GD Butcher I Heald CL Lee RJ Chambless LE Folsom AR Hirsch AT Dramaix M deBacker G Ankle brachial index combined with Framingham Risk Score to predict cardiovascular events and mortality: a meta-analysis JAMA 2008 300 2 197 208 18612117 10.1001/jama.300.2.197 PMC2932628

[B16] Elkeles RS Godsland IF Feher MD Rubens MB Roughton M Nugara F Humphries SE Richmond W Flather MD Coronary calcium measurement improves prediction of cardiovascular events in asymptomatic patients with type 2 diabetes: the PREDICT study Eur Heart J 2008 29 18 2244 2251 10.1093/eurheartj/ehn279 18573867

[B17] Lakoski SG Greenland P Wong ND Schreiner PJ Herrington DM Kronmal RA Liu K Blumenthal RS Coronary artery calcium scores and risk for cardiovascular events in women classified as "low risk" based on Framingham risk score: the multi-ethnic study of atherosclerosis (MESA) Arch Intern Med 2007 167 22 2437 2442 10.1001/archinte.167.22.2437 18071165

[B18] Lau KK Chan YH Yiu KH Tam S Li SW Lau CP Tse HF Incremental predictive value of vascular assessments combined with the Framingham Risk Score for prediction of coronary events in subjects of low-intermediate risk Postgrad Med J 2008 84 989 153 157 10.1136/pgmj.2007.064089 18372487

[B19] Murphy TP Dhangana R Pencina MJ D'Agostino RB Ankle-brachial index and cardiovascular risk prediction: an analysis of 11,594 individuals with 10-year follow-up Atherosclerosis 2012 220 1 160 167 10.1016/j.atherosclerosis.2011.10.037 22099055

[B20] Polak JF Pencina MJ Pencina KM O'Donnell CJ Wolf PA D'Agostino RB Carotid-wall intima-media thickness and cardiovascular events N Engl J Med 2011 365 3 213 221 10.1056/NEJMoa1012592 21774709 PMC3153949

[B21] Greenland P LaBree L Azen SP Doherty TM Detrano RC Coronary artery calcium score combined with Framingham score for risk prediction in asymptomatic individuals JAMA 2004 291 2 210 215 10.1001/jama.291.2.210 14722147

[B22] Kavousi M Elias-Smale S Rutten JH Leening MJ Vliegenthart R Verwoert GC Krestin GP Oudkerk M de Maat MP Leebeek FW Evaluation of newer risk markers for coronary heart disease risk classification: a cohort study Ann Intern Med 2012 156 6 438 444 22431676 10.7326/0003-4819-156-6-201203200-00006

[B23] Yoshida M Mita T Yamamoto R Shimizu T Ikeda F Ohmura C Kanazawa A Hirose T Kawamori R Watada H Combination of the framingham risk score and carotid intima-media thickness improves the prediction of cardiovascular events in patients with type 2 diabetes Diabetes Care 2012 35 1 178 180 10.2337/dc11-1333 22028278 PMC3241317

[B24] Alberti KG Zimmet PZ Definition, diagnosis and classification of diabetes mellitus and its complications. Part 1: diagnosis and classification of diabetes mellitus provisional report of a WHO consultation Diabet Med 1998 15 7 539 553 10.1002/(SICI)1096-9136(199807)15:7<539::AID-DIA668>3.0.CO;2-S 9686693

[B25] Yue WS Lau KK Siu CW Wang M Yan GH Yiu KH Tse HF Impact of glycemic control on circulating endothelial progenitor cells and arterial stiffness in patients with type 2 diabetes mellitus Cardiovasc Diabetol 2011 10 113 10.1186/1475-2840-10-113 22185563 PMC3258289

[B26] Whitworth JA 2003 World Health Organization (WHO)/International Society of Hypertension (ISH) statement on management of hypertension J Hypertens 2003 21 11 1983 1992 14597836 10.1097/00004872-200311000-00002

[B27] Executive summary of the third report of The National Cholesterol Education Program (NCEP) expert panel on detection, evaluation, and treatment of high blood cholesterol in adults (Adult Treatment Panel III) JAMA 2001 285 19 2486 2497 10.1001/jama.285.19.2486 11368702

[B28] Levey AS Bosch JP Lewis JB Greene T Rogers N Roth D A more accurate method to estimate glomerular filtration rate from serum creatinine: a new prediction equation. Modification of diet in renal disease study group Ann Intern Med 1999 130 6 461 470 10075613 10.7326/0003-4819-130-6-199903160-00002

[B29] Tsuchikura S Shoji T Kimoto E Shinohara K Hatsuda S Koyama H Emoto M Nishizawa Y Brachial-ankle pulse wave velocity as an index of central arterial stiffness J Atheroscler Thromb 2010 17 6 658 665 10.5551/jat.3616 20467192

[B30] Touboul PJ Hennerici MG Meairs S Adams H Amarenco P Bornstein N Csiba L Desvarieux M Ebrahim S Fatar M Mannheim carotid intima-media thickness consensus (2004–2006). An update on behalf of the Advisory Board of the 3rd and 4th Watching the Risk Symposium, 13th and 15th European Stroke Conferences, Mannheim, Germany, 2004, and Brussels, Belgium, 2006 Cerebrovasc Dis 2007 23 1 75 80 10.1159/000097034 17108679

[B31] Yiu KH Mok MY Wang S Ooi GC Khong PL Lau CS Tse HF Prognostic role of coronary calcification in patients with rheumatoid arthritis and systemic lupus erythematosus Clin Exp Rheumatol 2012 30 3 345 350 22409930

[B32] Wu Y Liu X Li X Li Y Zhao L Chen Z Rao X Zhou B Detrano R Liu K Estimation of 10-year risk of fatal and nonfatal ischemic cardiovascular diseases in Chinese adults Circulation 2006 114 21 2217 2225 10.1161/CIRCULATIONAHA.105.607499 17088464

[B33] Bernard S Serusclat A Targe F Charriere S Roth O Beaune J Berthezene F Moulin P Incremental predictive value of carotid ultrasonography in the assessment of coronary risk in a cohort of asymptomatic type 2 diabetic subjects Diabetes Care 2005 28 5 1158 1162 10.2337/diacare.28.5.1158 15855582

[B34] Rana JS Gransar H Wong ND Shaw L Pencina M Nasir K Rozanski A Hayes SW Thomson LE Friedman JD Comparative value of coronary artery calcium and multiple blood biomarkers for prognostication of cardiovascular events Am J Cardiol 2012 109 10 1449 1453 10.1016/j.amjcard.2012.01.358 22425333

[B35] Raggi P Shaw LJ Berman DS Callister TQ Prognostic value of coronary artery calcium screening in subjects with and without diabetes J Am Coll Cardiol 2004 43 9 1663 1669 10.1016/j.jacc.2003.09.068 15120828

[B36] Wong ND Nelson JC Granston T Bertoni AG Blumenthal RS Carr JJ Guerci A Jacobs DR Kronmal R Liu K Metabolic syndrome, diabetes, and incidence and progression of coronary calcium: the Multiethnic Study of Atherosclerosis study JACC Cardiovasc Imaging 2012 5 4 358 366 10.1016/j.jcmg.2011.12.015 22498324 PMC3327555

[B37] Chu ZG Yang ZG Dong ZH Zhu ZY Peng LQ Shao H He C Deng W Tang SS Chen J Characteristics of coronary artery disease in symptomatic type 2 diabetic patients: evaluation with CT angiography Cardiovasc Diabetol 2010 9 74 10.1186/1475-2840-9-74 21067585 PMC2992482

[B38] Gomez-Marcos MA Recio-Rodriguez JI Patino-Alonso MC Agudo-Conde C Gomez-Sanchez L Rodriguez-Sanchez E Gomez-Sanchez M Garcia-Ortiz L Yearly evolution of organ damage markers in diabetes or metabolic syndrome: data from the LOD-DIABETES study Cardiovasc Diabetol 2011 10 90 10.1186/1475-2840-10-90 21999369 PMC3214163

